# The plasticity of germ cell cancers and its dependence on the cellular microenvironment

**DOI:** 10.1111/jcmm.13082

**Published:** 2017-02-28

**Authors:** Daniel Nettersheim, Hubert Schorle

**Affiliations:** ^1^ Department of Developmental Pathology Institute of Pathology University Medical School Bonn Germany

**Keywords:** germ cell cancer, germ cell tumour, carcinoma *in situ*, germ cell neoplasia *in situ*, seminoma, embryonal carcinoma, cellular plasticity, microenvironment, reprogramming, SOX2

## Abstract

So far, the understanding of germ cell cancer (GCC) pathogenesis is based on a model, where seminomas and non‐seminomas represent distinct entities although originating from a common precursor termed germ cell neoplasia *in situ* (GCNIS). Embryonal carcinomas (ECs), the stem cell population of the non‐seminomas, is pluri‐ to totipotent and able to differentiate into cells of all three germ layers, giving rise to teratomas or tumours mimicking extraembryonic tissues (yolk sac tumours, choriocarcinomas). With regard to gene expression, (epi)genetics and histology, seminomas are highly similar to GCNIS and primordial germ cells, but limited in development. It remains elusive, whether this block in differentiation is controlled by cell intrinsic mechanisms or by signals from the surrounding microenvironment. Here, we reviewed the recent literature emphasizing the plasticity of GCCs, especially of seminomas. We propose that this plasticity is controlled by the microenvironment, allowing seminomas to transit into an EC or mixed non‐seminoma and vice versa. We discuss several mechanisms and routes of reprogramming that might be responsible for this change in the cell fate. We finally integrate this plasticity into a new model of GCC pathogenesis, allowing for an alternative view on the dynamics of GCC development and progression.

## Germ cell cancer pathogenesis

A defective primordial germ cell (PGC) development is thought to be the origin of a lesion termed GCNIS, which itself is the precursor of testicular type II GCCs 1, 2, 3, 4, 5, 6. GCCs can be subdivided into seminomas and non‐seminomas 3. With regard to gene expression, epigenetics and histology, seminomas are highly similar to GCNIS and PGCs. In contrast, ECs, the stem cell population of the non‐seminomas, are often described as a malignant counterpart to embryonic stem cells (ESC), showing features of pluri‐ to totipotency 3, 7. Thus, ECs are able to differentiate into cells of all three germ layers (teratomas) or extraembryonic tissues (yolk sac tumours, choriocarcinomas). GCCs commonly show amplification of the short arm of chromosome 12 (‘12p gain’), which is usually not detected in GCNIS 8, 9. This locus encodes for pluripotency and germ cell‐associated genes, like *NANOG*,* STELLA*,* BCAT1* and *GDF3* 10, 11.

In clinics, GCCs present as pure or mixed tumours. The finding that seminoma and GCNIS cells are highly similar to each other and ECs are more closely related to ESCs led to the hypothesis that formation of a seminoma is the default developmental pathway for GCNIS cells and that ECs develop through reprogramming of GCNIS cells 3. Furthermore, GCNIS cells might progress first into a seminoma, which becomes reprogrammed into a non‐seminoma (EC) later 3, 12, 13, 14, 15. It remains an open question whether GCNIS cells are able to develop into a seminoma and EC simultaneously.

## The microenvironment

The testis is an immune‐privileged organ, where a blood–testis barrier shields the germ cells from harmful outer effects, allowing for the generation of healthy sperm and faithful transmission of the genetic material to the next generation. GCCs normally grow within this microenvironment until they either penetrate the testis confines during invasive growth or spread to other sites in the body. Generally, GCCs metastasize along the body mid‐line via the lymphatic vessels, giving rise to GCCs in the brain, where they are termed germinomas (a seminoma‐like tumour) 3. Thus, during development, progression and metastasis, GCCs are confronted with different cellular microenvironments.

In general, a microenvironment is a specialized and isolated biophysical entity that influences its surroundings by specific signalling molecules, mitogens, enzymes, hormones, etc., provided by the cells growing within. The tumour microenvironment is defined as the specific location in which tumour cells exist and interact with their surrounding environment. It includes blood vessels, immune cells, fibroblast, lymphocytes and an extracellular matrix 16, 17, 18, 19. The crosstalk between the microenvironment and tumour cells has been extensively studied within the last years, demonstrating that tumour cells are able to influence their microenvironment and vice versa 16, 17, 19, 20.

Seminoma cells quickly initiate cell death upon disruption of their microenvironment in a process termed ‘anoikis’, suggesting that survival of seminomas critically depends on the microenvironment 21, 22. Furthermore, stromal cells surrounding tumour cells not only trigger tumour growth but also enhance the process of metastasis, whereas different cell types within the bone marrow microenvironment contribute to tumour‐induced bone disease 23, 24, 25. Tumour budding, which reflects invasiveness, metastasis and unfavourable prognosis in colorectal cancer, was associated with elements of the tumour microenvironment 26. So, there is a huge body of evidence that the microenvironment influences the behaviour, development and progression of cancer cells.

GCNIS and seminomas seem to be limited in their developmental capacity. It remains elusive, whether this block in differentiation is controlled by cell intrinsic mechanisms or by signals from the surrounding microenvironment.

## The developmental potential and plasticity of seminomas (TCam‐2)

Cellular plasticity describes the ability of cells to undergo a transition into another cell type, thereby fully adapting the newly acquired cell fate 27, 28. Most studies analysing the plasticity of seminomas are based on *in vitro* and *in vivo* experiments utilizing the seminoma‐derived cell line TCam‐2. TCam‐2 is the only available cell line, which reliably resembles a seminoma / GCNIS / PGC *in vitro* 29, 30, 31, 32, 33, 34, whereas the suitability of the cell lines JKT‐1 and SEM1 as a proxy for seminomas is questionable, although they were derived from tumours of patients diagnosed with seminoma 30, 35, 36.

Like seminomas and similar to ECs, TCam‐2 cells express pluripotency markers, like *OCT3/4*,* NANOG* and *LIN28*, but lack expression of the core pluripotency factor *SOX2* 30, 37. Instead, TCam‐2 / seminomas express the PGC specifier *SOX17* 30, 34, 37. *In vitro*, TCam‐2 is resistant to many differentiation‐inducing stimuli, like all‐trans‐retinoic acid (ATRA), the demethylating agent 5‐aza‐2‐deoxycytidine (5aza), the monoaminooxidase inhibitor tranylcypromine or a combination of all three 38, 39. Even a RNAi‐mediated knockdown of the pluripotency factor *NANOG* and the seminoma / PGC marker *TFAP2C* had no differentiation‐inducing effect 38, 40. So like seminomas, TCam‐2 cells are able to efficiently protect their seminoma‐like nature against differentiation‐inducing stimuli. In contrast, EC cells differentiate into cells of all three germ layers in response to ATRA or upon knockdown of *NANOG* expression 38, 39. Thus, although ECs display naïve / primed pluripotency allowing for differentiation, seminomas / TCam‐2 rather show a dormant pluripotency, meaning that they express pluripotency factors, but do not differentiate.

Orthotopic injection of TCam‐2 cells into the seminiferous tubules of the murine testis leads to a GCNIS‐ / seminoma‐like growth. However, TCam‐2 cells reprogramme into an EC‐like fate after transplantation into the murine flank or brain 33, 41. This clearly demonstrates that the microenvironment influences the seminoma (TCam‐2) fate and suggests that no further mutation is necessary for development of an EC from a seminoma.

## The molecular mode of action of the plasticity

In observing this remarkable and fast reprogramming of TCam‐2 cells, the molecular mechanisms had to be determined. It was obvious to look at the activity of receptors and their signalling molecules first. Interestingly, these studies revealed that BMP (Bone Morphogenetic Protein) signalling is inhibited after transplantation into the flank. In consequence, this leads to up‐regulation of *SOX2* and down‐regulation of *SOX17*. SOX2 triggers the induction of typical EC, pluripotency and epigenetic reprogramming factors, like *GDF3*,* DPPA3*,* NODAL*,* ZIC3* and *ZFP42* (*REX1*), whereas PGC / GCNIS / seminoma markers are down‐regulated (*SOX17*,* PRAME*,* cKIT*,* PRDM1*) 41. Additionally, NODAL signalling is induced 41, 42. Thus, inhibition of BMP signalling during the reprogramming to an EC reflects the loss of the seminoma‐ / PGC‐like character of TCam‐2.

Of note, DNA methylation levels strongly increase during the reprogramming, but the changes in DNA methylation follow the deregulations in gene expression 41, 43. So, the increased DNA methylation rather seems to reinforce the acquired EC‐like cell fate instead of being responsible for initiation of the reprogramming process.

The role of SOX2 in the reprogramming of TCam‐2 was tested by a loss‐of‐function approach. SOX2‐deficient TCam‐2 cells, when being xenografted into the flank of nude mice, do not undergo this reprogramming 42. They maintain a seminoma‐like morphology, global DNA methylation profile and gene expression signature 42. Although BMP signalling is also inhibited in SOX2‐deficient cells *in vivo*, the NODAL signalling cascade remains inactive 42. It has been demonstrated that SOX2 is responsible for establishment of the NODAL signalling loop by regulating expression of its cofactors *LEFTY1*/*2* and *CRIPTO*, but not *NODAL* itself 42. *In vitro*, treatment of TCam‐2 cells with the BMP signalling inhibitor NOGGIN led to up‐regulation of *SOX2* 41. Thus, *SOX2* must be induced upon repression of BMP signalling. Treatment of TCam‐2 (no *SOX2* expression) with recombinant NODAL did not lead to establishment of the NODAL signalling loop 41. So, SOX2 is required to activate NODAL signalling. In conclusion, the cells of the somatic microenvironment suppress BMP signalling, leading to derepression of *SOX2* and establishment of the NODAL signalling cascade. Thus, SOX2 is the driving force behind the reprogramming of seminomas to an EC‐like state.

Recently, Kushwaha *et al*. demonstrated that in TCam‐2 cells, *SOX2* is repressed by the polycomb repressive complex and the H3K27me3 chromatin mark enriched at the promotor 44. Future studies on TCam‐2 cells will have to show whether these repressive marks are lost during the *in vivo* reprogramming, whether BMP signalling is involved in establishment of these marks *in vitro* and whether these regulatory mechanisms can also be found in seminoma tissues.

It has been shown that PGCs / seminomas / TCam‐2 cells (SOX17 +) express the cancer/testis‐antigen *PRAME*, whereas non‐seminomas lack *PRAME* expression (SOX17 ‐) 39, 45. Additionally, *PRAME* is down‐regulated during *in vivo* reprogramming of TCam‐2 cells into an EC 41. So, *PRAME* expression correlates to *SOX17* expression and can be associated with a PGCs / seminoma cell fate. It has been proposed that PRAME regulates the pluripotency programme in seminomas / TCam‐2 cells and represses somatic and germ cell‐like differentiation processes by acting downstream of SOX17 39. Thus, SOX17 / PRAME is critically important for maintenance of an undifferentiated dormant pluripotent seminoma fate.

*In vivo* a subpopulation of SOX2‐deficient cells initiated differentiation into a cell type resembling a mixed non‐seminoma indicated by up‐regulation of germ layer differentiation markers *HAND1*,* PAX6*,* CDX1* and *FOXA2*, the trophoblast stem cell / choriocarcinoma marker *EOMES* and the yolk sac tumour marker *AFP* 42. This was reminiscent to the results of an *in vitro* differentiation of TCam‐2 into a mixed non‐seminoma 46. Therefore, the cells were forced to differentiate by cultivating the cells in murine fibroblast conditioned medium supplemented with FGF4 and heparin, which mimics a somatic microenvironment *in vitro* 46. Interestingly, a SOX2‐positive EC intermediate was skipped during the *in vitro* differentiation 46. These studies suggest that seminomas are also able to differentiate into a mixed non‐seminoma, but skip an EC intermediate. So, it seems that SOX2 is required for reprogramming of seminomas into an EC, but dispensable for a direct differentiation into a mixed non‐seminoma.

Further studies have to identify the factors that drive the development of seminomas into a mixed non‐seminoma. An interesting candidate gene is *FOXA2*, which is up‐regulated during the differentiation of TCam‐2 into a mixed non‐seminoma and was predicted to interact with many differentiation markers, such as *AFP*,* HAND1* and *EOMES* 42. FOXA2 is a pioneer factor able to open compacted chromatin and regulate gene expression in differentiated tissues and during embryonic development 47, 48. So far, the factors / events triggering up‐regulation of *FOXA2* during the differentiation of TCam‐2 into a mixed non‐seminoma are unknown.

## The plasticity of non‐seminomas

The studies described so far indicate that the microenvironment seems to be able to trigger reprogramming of seminomas into a pluripotent EC or directly into a mixed non‐seminoma. In consequence, but not proven yet, a transition of ECs into a seminoma seems likely. Future studies have to identify factors able to drive reprogramming of ECs into a seminoma‐like state. Recently, Irie *et al*. derived human PGC‐like cells from ESCs cultured in ‘4i’ medium (+ GSK3, MEK, P38‐kinase, JNK inhibitor, TGF‐b1, bFGF), rendering the ESCs germ cell competent 34. Thus, inhibition of WNT, MAPK and JNK signalling, while stimulating TGF and FGF signalling in parallel, initiates PGC‐like specification from ESCs. Additionally, Irie *et al*. identified SOX17, which acts upstream of BLIMP1 and TFAP2C, as a critical specifier of the PGC fate 34. In contrast, SOX2 was strongly down‐regulated in PGC‐like cells. SOX17 is expressed in GCNIS and seminomas / TCam‐2 (SOX2−), but not in ECs / ESCs (SOX2+) 37, 49. Assuming that ECs are highly similar to ESCs and that seminomas (TCam‐2) resemble PGCs 30, 34, the same molecular mechanisms allowing derivation of PGCs from ESC might be able to drive reprogramming of ECs into a seminoma. Thus, cultivation of EC cells / cell lines in ‘4i’ medium, while overexpressing SOX17 simultaneously, might trigger reprogramming of ECs to a seminoma.

Reprogramming of teratomas back to an EC‐like state or even seminoma‐like state seems unlikely due to the terminally differentiated nature of mature teratomas. Choriocarcinomas consist of cells resembling syncytio‐ and cytotrophoblast cells, which derive from cells of the extraembryonic trophectoderm. These extraembryonic trophectoderm / trophoblast cells can be reprogrammed to pluripotent stem cells by introducing the four Yamanaka factors (OCT3/4, SOX2, KLF4, cMYC) 50. Thus, reprogramming of choriocarcinoma cells to a stem cell‐like state seems technically possible, but remains elusive.

## An alternative model of germ cell cancer development

**Figure 1:**
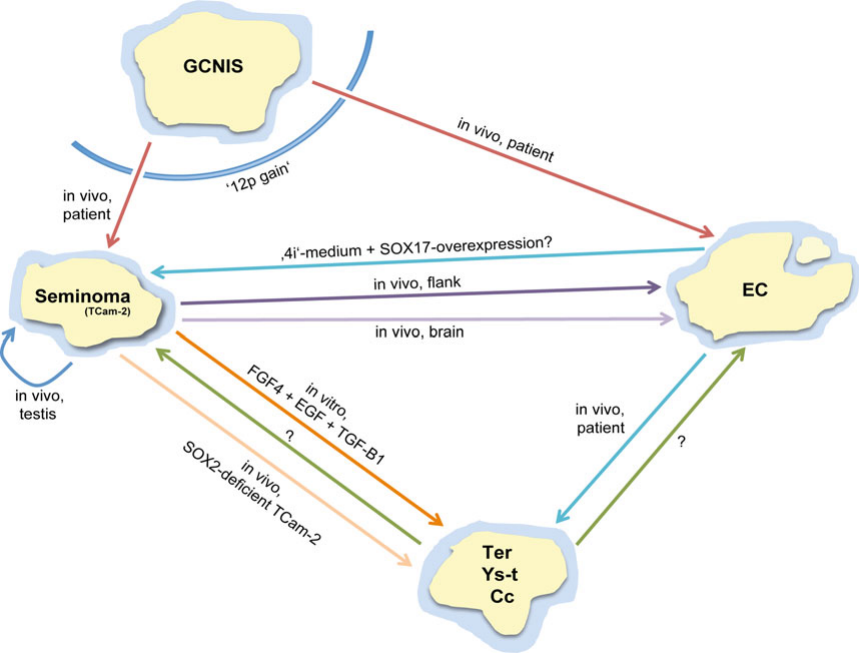
The dynamics of GCC development. New model of the dynamics of GCC development based on Sieweke's analogy of reprogramming processes to James Cook's journeys. Each island represents a GCC entity, which can be reached by ship if coordinates (*e.g*. culture conditions, reprogramming factors) and routes (*e.g*. developmental pathways) are known to the navigator. Sometimes more than one route is possible and other routes remain uncharted, yet (?). Once left, returning to an island might be prevented (‘12p gain’). GCNIS, germ cell neoplasia *in situ*; EC, embryonal carcinoma; Ter, teratoma; Ys‐t, yolk sac tumour; Cc, choriocarcinoma.

Based on the studies described in this article and on Sieweke's analogy of reprogramming processes as a map of James Cook's journeys, we propose an alternative view on the development of GCCs (Fig. 1) 51. There, each island represents a different GCC entity and if coordinates (*e.g*. culture conditions, reprogramming factors, mitogens) or routes (*e.g*. signalling pathways) are known, each island can be reached. Sometimes more than one route is available, some routes or islands remain uncharted and returning to an island might be (im)possible. Compared to the existing model of GCC pathogenesis, our model reflects the plasticity and dynamics of GCC development more accurately.

## Summary, conclusion and outlook

In summary, the development of the type II GCC entities seems to be a highly plastic process strongly influenced by the cellular microenvironment, allowing reprogramming of seminomas into a pluripotent EC or direct differentiation into a mixed non‐seminoma. Importantly, no additional genetic aberration, like a mutation seems to be necessary for switching the cell fate of GCCs.

The initial progression of GCCs from GCNIS relies on acquiring the ‘12p gain’ (by mutation), rendering the cells more aggressive. The ‘12p gain’ might also be the reason why seminomas and ECs cannot revert back to the GCNIS fate (Fig. 1). So once developing into a post‐GCNIS‐state by acquiring the ‘12p gain’, the microenvironment dictates the fate of GCCs.

These findings might also have implications for the therapy of GCCs. Patients initially diagnosed with a seminoma might develop an EC or mixed non‐seminoma during invasive growth or metastasis, when the tumour cells are confronted with a different microenvironment. As ECs grow more aggressive and require a harsher treatment than seminomas, an adaptation of the therapeutic strategy might be necessary to avoid an ineffective therapy.

Future studies should address whether ECs are able to adopt a seminoma cell fate, which factors drive the differentiation of seminomas into a mixed non‐seminoma, or whether reprogramming of a choriocarcinoma / yolk sac tumour to an EC might be possible. Also, albeit demonstrated in a murine xenotransplantation model, it remains elusive whether such reprogramming processes occur *in vivo*.

## Conflict of interest statement

The authors confirm that there are no conflicts of interest.
